# Survival analysis of increases in care needs associated with dementia and living alone among older long-term care service users in Japan

**DOI:** 10.1186/s12877-017-0555-8

**Published:** 2017-08-15

**Authors:** Huei-Ru Lin, Tetsuya Otsubo, Yuichi Imanaka

**Affiliations:** 1 The Japan Foundation for Aging and Health, 4F, 1-1 Aza Gengoyama, Oaza Morioka, Higashiura-cho, Chita-gun, Aichi 470-2101 Japan; 2 Department of Healthcare Economics and Quality Management, Graduate School of Medicine, Kyoto University, Yoshida Konoe-cho, Sakyo-ku, Kyoto, 606-8501 Japan

**Keywords:** Dementia, Long-term care, Living alone, Survival analysis, Care needs certification

## Abstract

**Background:**

Japan is known for its long life expectancy and rapidly aging society that there are various demands of older adults need to be fulfilled with, and one of them is long-term care needs. Therefore, Japan implemented the Long-Term Care Insurance in year 2000 for citizens who are above 65-year old and citizens who are above 40-year old in needs of long-term care services. This study was undertaken to longitudinally examine the influence of dementia and living alone on care needs increases among older long-term care insurance service users in Japan.

**Methods:**

Long-term care insurance claims data were used to identify enrollees who applied for long-term care services between October 2010 and September 2011, and subjects were tracked until March 2015. A Kaplan-Meier survival analysis was conducted to examine increases in care needs over time in months. Cox regression models were used to examine the effects of dementia and living alone on care needs increases.

**Results:**

The cumulative survival rates before care needs increased over the 4.5-year observation period were 17.6% in the dementia group and 31.9% in the non-dementia group. After adjusting for age, sex, care needs level, and status of living alone, the risk of care needs increases was found to be 1.5 times higher in the dementia group. Living alone was not a significant risk factor of care needs increases, but people with dementia who lived alone had a higher risk of care needs increases than those without dementia.

**Conclusion:**

Dementia, older age, the female sex, and lower care needs levels were associated with a higher risk of care needs increases over the study period. Among these variables, dementia had the strongest impact on care needs increases, especially in persons who lived alone.

## Background

It has been more than 15 years since the long-term care (LTC) insurance system was implemented in Japan in 2000. Japan is known for its long life expectancy and rapidly aging society. In 2015, older adults aged 65 years or older and 75 years or older account for 26.8% and 13.0% of the total population, respectively, and these proportions have increased approximately 0.3% per year [1].

The LTC insurance system covers all citizens aged 40 years and older in Japan. From the age of 40 years, citizens are obligated to pay LTC insurance premiums and become insured. The managing entities (insurers) of the LTC insurance system are the municipal governments. The national and prefectural governments provide support to the municipalities to ensure effective implementation of the LTC insurance system.

Once an insured person applies to use any LTC service, they will first be certified at SRLs 1 to 2 or CNLs 1 to 5 by the various Kyoto municipal governments. The relevant LTC approval board in each municipality assesses the physical and mental condition of individual applicants, and determines eligibility based on evaluations by a gatekeeper. The SRL categories represent a lower requirement for care than the CNL categories, and higher numbers for each level indicate increasing dependency and requirement for LTC services.

Insured individuals certified at SRL 1 or 2 receive long-term care preventive service planning to use home care services or community-based care services. These include visiting care, day care rehabilitation, and healthcare management guidance by nutritionists, nurses, dental hygienists, pharmacists, and physicians. Insured individuals certified at CNL 1 to 5 are eligible to use home care services, community-based care services, and facility care services (including short- and long-term stays in a facility). In principle, users must pay an out-of-pocket copayment of 10 to 20% of care service costs to service providers depending on their financial status.

Due to the accumulation of LTC insurance claims data and the nationwide coverage of this system, the potential research applications of these data have grown rapidly. In addition, the number of persons enrolled in the LTC insurance system is increasing every year as the older population expands. According to Japan’s Ministry of Health, Labour and Welfare, the number of LTC insurance enrollees in 2014 (33.0 million) was 1.5 times that of the number in 2000 (22.4 million) [2].

Traditionally, older adults in Japan have lived with their extended families and received informal care from family members. However, the nuclear family has become the prevalent family unit in modern society, and the number of older adults who live alone has inevitably increased [3]. Due to the lack of family support, older adults who live alone may be at a higher risk of future disability [4, 5] and substandard healthcare quality [6]. In persons with dementia, living alone has been identified as an unsafe activity [7], and is associated with a higher risk of unmet needs in both medical care and LTC [8]. However, a previous study found that people with dementia who live alone did not have more health risks than those living with others [9].

Several studies have reported that dementia has a strong impact on medical and LTC expenditure, service utilization, comorbidities, mortality, and changes to care needs [10–23]. Although Lin et al. [23]and Matsuda et al. [21] examined the association between dementia and care needs increases, those analyses utilized a cross-sectional approach. Other studies have been conducted on small or geographically-limited samples in special settings (such as home care service users), thereby resulting in non-representative samples [17–20]. Furthermore, previous survival analyses of LTC users were mostly conducted with a focus on mortality. For example, Choi and Joung confirmed the effect of LTC service use on mortality [16], and Koller et al. reported that dementia increased the risk of mortality by 1.5 times [15]. Although various determinants of changes in care needs have been identified, the time duration until care needs increases has yet to be examined. Moreover, the number of older adults with dementia and who live alone is increasing, but the possible influence of these factors on care needs increases among LTC insurance users in Japan remains unclear.

Hence, the aim of this study was to longitudinally examine the influence of dementia and living alone on care needs increases among older adults enrolled in the LTC insurance system in Japan.

## Methods

### Database

We collected data from Kyoto prefecture, Japan. The data comprised reimbursement claims electronically submitted from LTC service providers to municipalities (city, town, and village governments), which serve as insurance payers in Japan. These data also include LTC service details and claims for users’ activities, and are routinely collected from the long-term care service providers located in Kyoto prefecture.

### Design and study population

In this retrospective study, we used an LTC insurance claims dataset to identify individuals aged 65 years and older who had applied for LTC services and had been certified at Support Required Levels (SRLs) 1 to 2 or Care Needs Levels (CNLs) 1 to 4 between October 2010 and September 2011 in Kyoto Prefecture, Japan. Subjects were tracked until the end of March 2015.

Although LTC insurance users may also be certified at CNL 5 (representing the highest requirement for care needs at the baseline: 11,838), we excluded these subjects because we would not be able to identify further deterioration in their condition. The study sample comprised 77,159 subjects who had applied for LTC insurance during the initial registration year (October 1, 2010 to September 30, 2011).

### Covariates

Data on age and sex were collected during the LTC insurance registration month of each subject during the initial registration year. Subjects with dementia were identified through their use of dementia-related LTC services (e.g., extra benefits for dementia, use of dementia wandering alarms) during the observation period. The status of living alone was also identified using LTC insurance claims data because subjects who live alone are eligible for additional reimbursements under the LTC insurance system. Since this additional reimbursement is provided based on the living arrangements of the previous month, we examined if subjects were living alone or with others using data from September 1, 2010 to February 28, 2015. Care needs certification (SRLs and CNLs) was identified beginning from the LTC insurance registration month of each subject, and tracked on a monthly basis until March 2015. An increase in care needs (event) was defined as a change from SRL to CNL or an increase in the SRL or CNL categories.

### Statistical analysis

The main outcome measure was the number of months from LTC insurance registration to care needs increase. Follow-up began on the first month for each subject during the registration year, and ended either on the month of care needs increase or on March 31, 2015, whichever was earlier. First, we conducted a descriptive analysis of the subjects, and assessed the crude survival estimates using the Kaplan-Meier method stratified for dementia and non-dementia.

Next, Cox proportional hazard regression analyses were performed using the following 5 models to estimate hazard ratios (HRs) and 95% confidence intervals (95% CI). Model 1 was constructed using only dementia as an independent variable. Model 2 included age (four categories) and sex (two categories) as independent variables in addition to dementia. Model 3 included care needs certification (six categories) in addition to the independent variables in Model 2. Model 4 included the status of living alone or with others (two categories) in addition to the independent variables in Model 3. In order to elucidate the interaction between dementia and living alone, we also included a dementia-living alone interaction variable in Model 5. Additionally, we calculated the HR for each SRL and CNL to clarify the influence of dementia on the different categories.

All analyses were performed using IBM SPSS Statistics Version 23 (Release 23.0.0.2). *P* values (two-tailed) below 0.05 were considered statistically significant. This study was approved by the Ethics Committee of Kyoto University Graduate School of Medicine (R0438).

## Results

A total of 23,638 (30.6%) subjects with dementia and 53,521 (69.4%) subjects without dementia were analyzed. The baseline characteristics of LTC insurance users are shown in Table 1.

**Table 1:** Baseline characteristics of the study subjects

Baseline Characteristics	Total		Dementia		Non-Dementia		*P* value^a^
n (%)	77159	(100%)	23638	(30.6%)	53521	(69.4%)	
Age, mean (standard deviation)	83.48	(7.6)	84	(7.1)	83.26	(7.8)	<0.05
Age groups, n (%)							<0.05
65-74 y	13835	(17.9%)	3413	(14.4%)	10422	(19.5%)	
75-84 y	30814	(39.9%)	9629	(40.7%)	21185	(39.6%)	
85-94 y	31091	(40.3%)	10172	(43.0%)	20919	(39.1%)	
≥95 y	1419	(1.8%)	424	(1.8%)	995	(1.9%)	
Sex, n (%)							<0.05
Male	23884	(31.0%)	7019	(29.7%)	16865	(31.5%)	
Female	53275	(69.0%)	16619	(70.3%)	36656	(68.5%)	
Care needs, n (%)							<0.05
Support Required Level 1	1354	(1.8%)	353	(1.5%)	1001	(1.9%)	
Support Required Level 2	2959	(3.8%)	600	(2.5%)	2359	(4.4%)	
Care Needs Level 1	18065	(23.4%)	5409	(22.9%)	12656	(23.6%)	
Care Needs Level 2	22481	(29.1%)	6655	(28.2%)	15826	(29.6%)	
Care Needs Level 3	17747	(23.0%)	6257	(26.5%)	11490	(21.5%)	
Care Needs Level 4	14553	(18.9%)	4364	(18.5%)	10189	(19.0%)	
Living alone, n (%)							<0.05
Yes	14045	(18.2%)	9612	(18.0%)	4433	(18.8%)	
No	63114	(81.8%)	43909	(82.0%)	19205	(81.2%)	

^a^ *P* values were calculated using the chi-square test (categorical variables) and *t*-test (continuous variables) between the dementia group and non-dementia group

The mean age ± standard deviation (SD) was 83.48 ± 7.6 years for all subjects, 84 ± 7.14 years for the dementia group, and 83.26 ± 7.79 years for the non-dementia group. Most of the subjects were women (69.0%), and more than 80% were 75 years and older. The most common care needs certification was CNL 2 (29.1%), followed by CNL 1 (23.4%), CNL 3 (23%), CNL 4 (18.9%), SRL 2 (3.8%), and SRL 1 (1.8%). Approximately 18.2% of all subjects lived alone. The initial analysis showed significant differences for all characteristics between the dementia and non-dementia groups. The mean follow-up duration was 21.4 months (SD: 17.14) for all subjects, 20.4 months for the dementia group, and 21.9 months for the non-dementia group.

The cumulative survival rates before care needs increased in Years 1 to 4.5 are presented in Table 2. Throughout the observation period, our results showed that dementia, the female sex, older age, and lower care needs certifications had a higher proportion of care needs increases when compared with no dementia, the male sex, younger age, and higher care needs certifications, respectively. People who lived alone showed a higher proportion of care needs increases in Years 1 to 3, but the discrepancy between living alone and not living alone diminished and reversed in Years 4 and 4.5. The cumulative survival rates before care needs increased over the 4.5-year observation period were 17.6% in the dementia group and 31.9% in the non-dementia group. The female sex, older age, and lower care needs certifications were also indicative of a lower cumulative survival rate before care needs increased.

**Table 2:** The cumulative survival rates before care needs increased at Years 1, 2, 3, 4, and 4.5

	Year 1	Year 2	Year 3	Year 4	Year 4.5
Dementia					
Yes	63.4%	43.3%	29.2%	20.4%	17.6%
No	71.7%	56.3%	44.4%	35.5%	31.9%
Age					
65-74 y	72.6%	57.1%	46.2%	38.3%	35.1%
75-84 y	69.0%	52.5%	39.5%	30.5%	27.3%
85-94 y	67.6%	49.7%	36.3%	27.0%	23.4%
≥95 y	67.5%	49.0%	36.5%	25.5%	21.7%
Sex					
Female	68.6%	51.2%	38.3%	29.1%	25.7%
Male	69.9%	54.3%	41.6%	33.7%	30.6%
Care needs					
Support Required Level 1	0.0%	0.0%	0.0%	0.0%	0.0%
Support Required Level 2	0.0%	0.0%	0.0%	0.0%	0.0%
Care Needs Level 1	63.4%	43.8%	29.3%	20.8%	17.5%
Care Needs Level 2	73.8%	55.8%	42.1%	32.9%	29.5%
Care Needs Level 3	76.4%	58.0%	44.7%	34.9%	31.2%
Care Needs Level 4	82.3%	67.5%	56.5%	46.6%	43.0%
Living alone					
Yes	67.0%	51.5%	39.1%	30.4%	27.3%
No	69.5%	52.2%	39.3%	30.3%	26.9%
Overall	73.1%	58.3%	46.7%	38.4%	35.2%

Table 3 summarizes the mean time to care needs increases (mean number of months without care needs increases) and the half-deterioration period (interval of time in months required for half of the subjects to experience care needs increases).

**Table 3:** Mean time to care needs increases and median half-deterioration period

	Mean Time (Months)^a^	(95% CI)	Half-deterioration period (Months)^b^	(95% CI)
Dementia				
Yes	25.0	(24.8–25.3)	21.0	(20.6–21.4)
No	31.2	(31.1–31.4)	31.0	(30.5–31.5)
Sex				
Female	28.8	(28.6–28.9)	26.0	(25.6–26.4)
Male	30.3	(30.0–30.6)	30.0	(29.4–30.6)
Age				
65–74 y	29.3	(29.0–29.5)	27.0	(26.5–27.5)
75–84 y	28.0	(27.7–28.2)	25.0	(24.6–25.5)
85–94 y	27.8	(26.7–28.9)	24.0	(21.7–26.3)
≥95	29.2	(29.0–29.4)	27.0	(26.7–27.3)
Care needs				
Support Required Level 1	5.2	(5.1–5.4)	5.0	(4.8–5.2)
Support Required Level 2	5.5	(5.4–5.6)	5.0	(4.8–5.2)
Care Needs Level 1	25.1	(24.8–25.3)	20.0	(19.5–20.5)
Care Needs Level 2	30.9	(30.6–31.2)	30.0	(29.4–30.6)
Care Needs Level 3	32.1	(31.8–32.4)	31.0	(30.2–31.8)
Care Needs Level 4	36.8	(36.4–37.2)	46.0	(44.56–47.44)
Living Alone				
Yes	28.7	(28.3–29.1)	26.0	(25.2–26.8)
No	29.3	(29.1–29.5)	27.0	(26.7–27.3)
Overall	29.2	(29.0–29.4)	27.0	(26.7–27.3)

*Abbreviation*: *CI* confidence intervals

^a^The average number of months before care needs increased

^b^The interval of time in months required for half of the subjects to experience care needs increases

The dementia group had a mean of 25 months (95% CI: 24.77–25.28) without care needs increases, which was shorter than the 31.2 months (95% CI: 31.05–31.43) in the non-dementia group. Similarly, the half-deterioration period of the dementia group was 21 months (95% CI: 20.59–21.41), which was shorter than the 31 months (95% CI: 30.51–31.49) in the non-dementia group. When compared with men, women had shorter durations for both the mean time to care needs increases and half-deterioration period. With the exception of subjects aged 95 years and older, younger age was associated with reduced mean time to care needs increases and half-deterioration periods. In addition, the mean time to care needs increases and half-deterioration periods reduced as care needs certification decreased. Subjects who lived alone had shorter mean times to care needs increases and half-deterioration periods than those who lived with others. Overall, the mean time to care needs increases was 29.2 months and the half-deterioration period was 27 months.

**Fig. 1:**
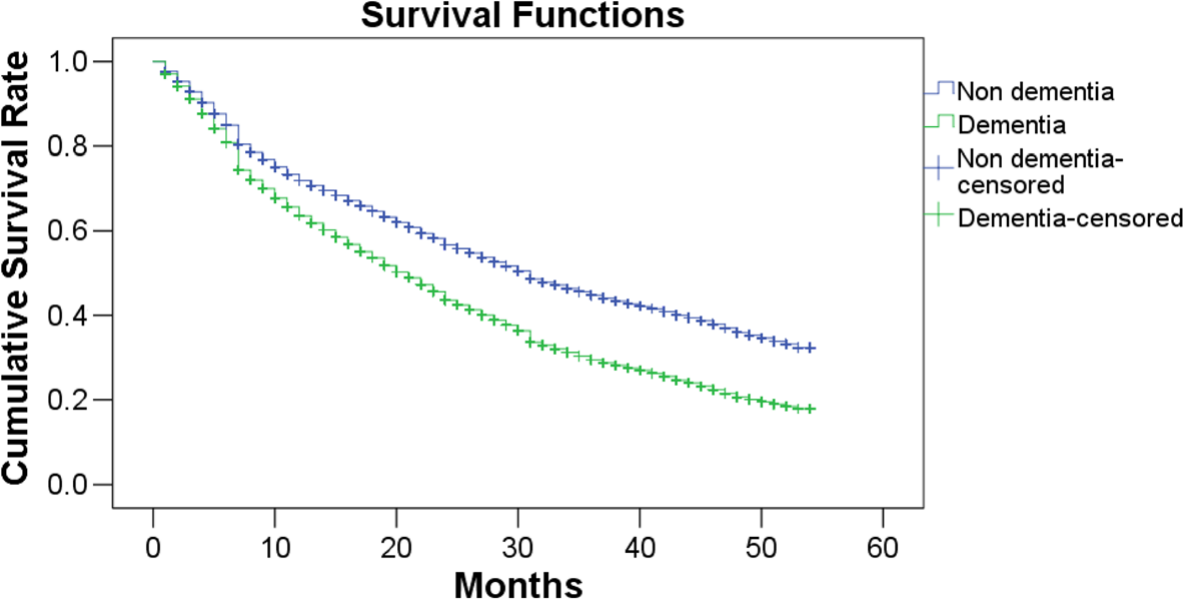
Survival analysis of dementia users and non-dementia users for increases in care needs. Follow-up began in the first month of using long-term care insurance services. *P* for log-rank test < 0.0001

Figure 1 shows the Kaplan-Meier survival curve of the time until care needs increases for the dementia and non-dementia groups.

**Fig. 2:**
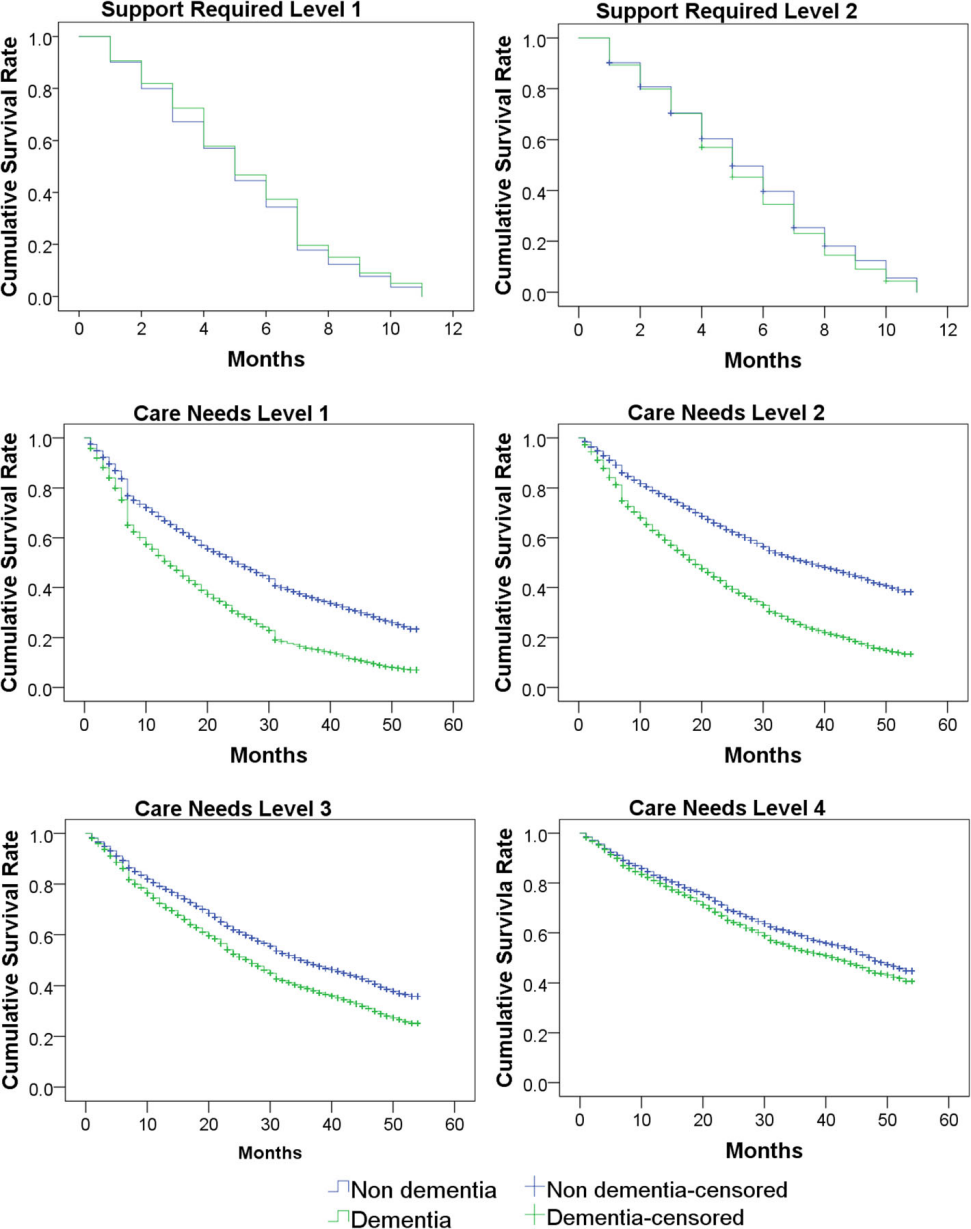
Survival analysis of dementia users and non-dementia users for increases in care needs according to the initial care needs certification. Follow-up began in the first month of using long-term care insurance services. All *P* for log-rank test < 0.0001

Although both curves decreased throughout the course of the follow-up period, the reduction was greater in the dementia group (*P* for log-rank test: <0.0001). Figure 2 shows the results of the analysis of care needs increases in the dementia and non-dementia groups according to each subject’s initial care needs certification.

As with the results of the overall subjects, both curves were observed to decrease over the observation period for all subgroups. The decrease was more severe in subjects with dementia for all CNL categories; however, this disparity was not observed in the SRL categories. The *P* values for the log-rank test were below 0.0001. The results showed that almost all of the subjects initially certified with SRL 1 and 2 experienced care needs increases within 12 months.

In order to control for the influence of various covariates of dementia on care needs increases, we constructed and analyzed 5 Cox proportional hazard regression models. The results are shown in Table 4.

**Table 4:** Cox regression analysis results of the factors associated with care needs increases

	Model 1		Model 2		Model 3		Model 4		Model 5	
Characteristic	HR	95% CI	HR	95% CI	HR	95% CI	HR	95% CI	HR	95% CI
Dementia(Ref: Non-Dementia)	1.46**	1.44–1.49	1.45**	1.42–1.48	1.55**	1.52–1.58	1.55**	1.52–1.58	1.53**	1.49–1.56
Age (Ref: 65-74 y)										
75-84 y			1.16**	1.13–1.20	1.06**	1.52–1.58	1.07**	1.04–1.10	1.07**	1.04–1.10
85-94 y			1.24**	1.21–1.28	1.20**	1.16–1.23	1.19**	1.15–1.22	1.19**	1.15–1.22
≥95 y			1.26**	1.17–1.36	1.37**	1.27–1.47	1.35**	1.25–1.45	1.35**	1.25–1.45
Female (Ref: Male)			1.07**	1.05–1.09	1.05**	1.03–1.07	1.06**	1.04–1.08	1.06**	1.04–1.08
Care needs (Ref: Care Needs Level 4)										
Support Required Level 1					12.65**	11.89–13.45	13.25**	12.46–14.10	13.26**	12.46–14.10
Support Required Level 2					12.27**	11.70–12.86	12.85**	12.25–13.49	12.87**	12.27–13.50
Care Needs Level 1					2.21**	2.14–2.29	2.30**	2.23–2.39	2.31**	2.23–2.38
Care Needs Level 2					1.54**	1.49–1.59	1.58**	1.53–1.63	1.59**	1.53–1.63
Care Needs Level 3					1.36**	1.31–1.41	1.37**	1.33–1.42	1.37**	1.33–1.42
Living Alone							0.84**	0.81–0.86	0.81**	0.78–0.83
Living Alone*Dementia									1.09*	1.04–1.15

*Abbreviations*: *CI* confidence intervals; *HR* hazard ratio

**P value < 0.001

*P value < 0.01

In Model 1, we analyzed the HRs for care needs increases while including only dementia status as an independent variable. The results showed that subjects with dementia had a higher risk of care needs increases throughout the observation period that is 1.46 times that of subjects without dementia. In Model 2, which incorporated age and sex, the risk of care needs increases rose together with increasing age (ref: 65–74 y; HR for 75–84 y: 1.16, 95% CI: 1.13–1.2; HR for 85–94 y: 1.24, 95% CI: 1.21–1.28; HR for ≥95 y: 1.26, 95% CI: 1.17–1.36), and women were associated with a higher risk of care needs increases (HR: 1.07, 95% CI: 1.05–1.09). After adjusting for age and sex, the dementia group still had a higher risk of care needs increases (HR: 1.45, 95% CI: 1.42–1.48) when compared with the non-dementia group. In Model 3, which included care needs certification, the effects of most of the other covariates decreased. However, the HR of dementia increased with the inclusion of care needs certification. In Model 4, which included the status of living alone, the results were similar to those of Model 3. Also, living alone was not a risk factor of care needs increases. In order to examine the interaction between dementia and living alone, we included an interaction factor in Model 5, and found that subjects with dementia who lived alone had a higher risk of care needs increases (HR: 1.09, 95% CI: 1.04–1.15).

The results of the Cox proportional hazard regression analyses for the various initial care needs certifications are presented in Table 5.

**Table 5:** Cox regression analysis results of the factors associated with care needs increases stratified for care needs certifications

	Support Required Level 1		Support Required Level 2		Care Needs Level 1		Care Needs Level 2		Care Needs Level 3		Care Needs Level 4	
Characteristic	HR	95% CI	HR	95% CI	HR	95% CI	HR	95% CI	HR	95% CI	HR	95% CI
Dementia(Ref: Non-Dementia)	0.93	0.80–1.08	1.14*	1.02–1.28	1.70**	1.63–1.77	1.92**	1.84–2.00	1.33**	1.27–1.39	1.17**	1.1–1.24
Age (Ref: 65-74 y)												
75-84 y	1.11	0.93–1.32	1.00	0.90–1.13	1.14**	1.08–1.21	1.07*	1.01–1.13	1.04	0.98–1.10	0.99	0.92–1.07
85-94 y	1.03	0.86–1.24	0.97	0.86–1.09	1.35**	1.28–1.43	1.25**	1.19–1.32	1.10*	1.03–1.16	1.02	0.95–1.10
≥95 y	1.19	0.65–2.21	0.93	0.65–1.35	1.64**	1.40–1.92	1.55**	1.36–1.76	1.25*	1.08–1.45	0.95	0.79–1.14
Female (Ref: Male)	0.99	0.88–1.11	0.94	0.86–1.02	0.94*	0.90–0.98	1.05*	1.01–1.09	1.17**	1.11–1.23	1.32**	1.23–1.41
Living Alone	0.96	0.84–1.10	1.02	0.93–1.11	0.85**	0.81–0.89	0.76**	0.71–0.81	0.75**	0.68–0.84	0.70**	0.58–0.85
Living Alone*Dementia	1.03	0.79–1.33	0.89	0.73–1.07	1.05	0.97–1.14	1.12*	1.02–1.23	1.15	1.00–1.32	1.15	0.89–1.49

*Abbreviations*: *CI*confidence intervals; *HR* hazard ratio

**P value < 0.001

*P value < 0.01

In the models for both SRLs, all independent variables, with the exception of dementia in SRL 2, were not significantly associated with care needs increases. Among the models for CNLs 1 to 4, the highest HR for dementia was observed in CNL 2 (HR: 1.95, 95% CI: 1.88–2.02). In addition, the impact of dementia on care needs increases decreased from CNL 2 with increasing care needs certifications. Older age was associated with care needs increases except in CNL 4. Women had a higher risk of deterioration than men, and this risk rose with increasing care needs certifications. Furthermore, living alone was significantly associated with a lower risk of care needs increases in CNLs 1 to 4, but not in SRL 1 and 2.

## Discussion

In this study, we analyzed 23,638 LTC insurance users with dementia and 53,521 users without dementia to investigate the influence of dementia and living alone on care needs increases in older adults over a period of 4.5 years. Among the variables analyzed for care needs certification, dementia had the strongest impact on care needs increases, even among insured persons who lived alone.

Our findings corroborated those of previous studies [24, 25] that reported a lack of association between living alone and functional decline. In fact, older adults who are able to live alone may actually have higher functional ability [25, 26]. However, some studies report that living alone is significantly associated with an increased risk of hospitalization and falls [27, 28]. Additionally, the lack of social support may bring about mental illness or accidents [5, 29, 30], and has a strong impact on the risk of future disability [4]. A previous study investigated psychological and mental factors separately, and noted that functional impairment increased significantly with mental impairment [31]. Furthermore, our analysis found that people living alone had a higher cumulative survival rate before care needs increased during Years 1 to 3 when compared with those living with others, but the discrepancy between the groups diminished and even reversed from Year 4 onward. Because living alone may imply higher physical function in some older adults, the ability to live alone for a long period may actually be indicative of higher levels of independence. Nevertheless, measures to prevent care needs increases should not only consider the physical functional ability of older adults, but also support their mental stability.

Although living alone was not previously found to be a health risk factor, persons with dementia who live alone are at increased risk for unmet medical, psychological, social, and environmental needs, and also tend to utilize more services than persons without dementia [8, 9, 21]. With changes in household composition, the proportion of people living alone has increased over time [31, 32]. There should therefore be a focus on the LTC needs of older adults, especially those who live alone. As mental health is an evaluation criterion for care needs certification, cognitive impairment may explain why persons with dementia who live alone would have a higher risk for care needs increases. Therefore, social support may be particularly important for preventing cognitive impairment [6, 33].

We demonstrated that dementia is a strong risk factor of care needs increases even after controlling for age, sex, care needs certification, and living alone. This finding corresponds to those of previous studies on care needs increases [23] and functional decline in activities of daily living [11]. In addition, the half-deterioration period of the dementia group was 21 months, which was shorter than the 31 months of the non-dementia group. This suggests that dementia is not only associated with care needs increases, but may also accelerate the speed of increase. The half-deterioration periods for older age, the female sex, lower care needs certifications, and living alone were shorter than those for younger age, the male sex, higher care needs certifications, and not living alone, respectively. With the exception of care needs certifications, the differences for all these variables were smaller than those between the dementia and non-dementia groups. Although dementia was not significantly associated with care needs increases for the SRL1, it contributed to a higher risk in subjects with SRL 1 and CNLs 1 to 4 (especially in the lower CNL categories).

The renewal of SRL certification is carried out 3 to 24 months after the initial certification. Most subjects with SRL are likely to be reassessed within 12 months (average of 6 months), and may be re-certified as no longer requiring care or having higher care needs. Concurrently, the HRs in the care needs certification-stratified analysis (Table 5) demonstrated the decreasing impact of dementia in subjects with CNLs but not SRLs. This suggests that other confounders, and not dementia itself, are contributing to care needs increases.

In subjects initially certified with CNLs 1 to 3, the curves showed sudden decreases around 6 to 8 months after registration (Fig. 2). This may be indicative of certification renewal for CNLs, which is conducted 6 to 24 months after the initial certification. Therefore, a large proportion of these subjects may receive a new CNL certification after 6 to 8 months. Moreover, in subjects with CNL 1, there was another sudden drop observed after approximately 30 months. Subjects who receive a CNL certification for the first time may be provisionally classified as CNL 1 while an appropriate care plan is developed. Following the first reassessment after 6 months, they may receive a more suitable CNL with a more appropriate care plan that is then sustained for the longest time period before reassessment, i.e., 24 months. This may explain the second sudden drop observed after 30 months.

A previous analysis also showed that dementia had a stronger impact on lower CNLs [23]. However, previous studies have generally excluded the SRL categories, and studies that addressed care needs increases in Japan have focused only on insured individuals with CNLs 1 to 5 or had merged CNLs into subgroups [17, 20, 23, 34]. In contrast, our present study included SRL, and found that dementia was not significantly associated with care needs increases in SRL 1. According to the Ministry of Health, Labour and Welfare, individuals certified at SRL 1 and 2 are generally able to live independently, but are at higher risk of requiring LTC in the future [1]. Consequently, dementia and other risk factors may contribute to care needs increases in individuals with SRL 1 and 2. In our study, almost all of the subjects with SRL 1 and 2 experienced increases in care needs during the first year of the observation period. The reassessment period for first time service users is 3 to 12 months (usually 6 months after the first certification). A number of users with SRL may be first time users of LTC services, and it is highly likely that our subjects with higher care needs certifications in the first year had undergone this scheduled reassessment. As SRL certification indicates a lower level of physical and mental functional decline, people with this level of care needs may return to normal life after undergoing rehabilitation or learning to live independently. Therefore, the underlying reason for why all the subjects with SRL in our study experienced increases in care needs in their first year should be further investigated.

Our findings indicate that subjects with higher CNLs had lower risk of increasing care needs. Since the reassessment period for LTC insurance users is 3 to 24 months, users with higher CNLs may have a longer history of using LTC services and a relatively stable status. On the other hand, users with lower CNLs may have a more unstable status, and therefore have a tendency to be re-certified after a shorter period of time. This may have manifested as a higher risk of physical and mental functional decline in these users.

In concordance with previous studies [17–19, 23], we found older age to be a risk factor of care needs increases, and its influence would increase in older subjects as they are more likely to develop functional impairments. Because physical and mental disabilities in older adults would affect functional ability in activities of daily living and instrumental activities of daily living [35–37], it is unsurprising that age was identified to be a risk factor of care needs increases.

With regard to sex, our analysis found that women had a higher risk of care needs increases when compared to men for all analytical models. This result is different from some previous studies that had been conducted using a cross-sectional approach or a relatively short-term (2 years) longitudinal approach [4, 34]. Sjolund et al. [36] suggested that women are more likely to live longer with diseases, resulting in higher levels of disability. However, another study reported that men tend to have higher functional recovery than women at the beginning of functional impairment [37]. Additionally, John et al. conducted a 5-year longitudinal study and reported that women had a higher risk of functional status impairment than men over a 2.5-year period and a 5-year period [38]. Because our study used a 4.5-year observation period, the timespan is similar to that of John et al. [38]; this may have accounted for the similar results regarding sex. Therefore, the differences in findings between our study and previous studies regarding sex may be due to the different observation periods or study design. The impact of time-sex interaction should be investigated in future research.

### Limitations

The major limitations of this study are similar to those that use claims data. First, our covariates are limited to variables that are available in the database. Therefore, we were unable to consider factors such as socioeconomic status, education level, or cultural factors. Furthermore, we were unable to trace subjects if they had died, moved to another city, or changed insurers during the observation period. Next, dementia was identified using only the dementia-associated LTC services provided to each subject. Although subjects who use these services are required to have a medical certificate issued by a physician, there may be a number of subjects with dementia who have not been treated by a physician or must use other services due to limited resources (e.g., communal daily LTC for dementia patient is limited by the number of beds). This may have led to an underestimation of the number of subjects with dementia in this study. Because dementia is difficult to diagnose, future studies should focus on more accurately identifying patients with dementia. In addition, subjects were identified as living alone using the additional reimbursement provided by the LTC insurance system. However, these reimbursements are provided based on the living status of the previous month, and therefore did not shed light on real-time changes to living arrangements.

In additional, the evaluation criteria for care needs certification include indicators of physical and mental function. However, we identified care needs increases using only changes to SRL and CNL certifications, and therefore could not distinguish between the influences of physical and mental function. As a result, this may have prevented the clear elucidation of the impact of living alone or dementia on care needs increases.

Finally, this study is an observational study, and therefore cannot elucidate the causal relationship between dementia and care needs increases. However, to the best of our knowledge, this large-scale study uses the longest observation period (4.5 years) so far in Japan.

## Conclusion

Our results revealed that dementia, older age, the female sex, and lower care needs certification were associated with a higher risk of care needs increases over a 4.5–year period. Dementia had the highest impact on care needs increases among all variables, especially in older adults living alone. Furthermore, the half- deterioration period may have applications as a quality indicator for use in the LTC insurance system.

## Abbreviations

CI: Confidence interval
CNL: Care Needs Levels
HR: Hazard ratios
LTC: Long-term care
SD: Standard deviation
SRL: Support required level
